# Intrinsic Differences between Oral and Skin Keratinocytes

**DOI:** 10.1371/journal.pone.0101480

**Published:** 2014-09-08

**Authors:** Anna Turabelidze, Shujuan Guo, Allison Yen Chung, Lin Chen, Yang Dai, Phillip T. Marucha, Luisa A. DiPietro

**Affiliations:** 1 The Richard and Loan Hill Department of Bioengineering, College of Engineering, University of Illinois at Chicago, Chicago, Illinois, United States of America; 2 Center for Wound Healing and Tissue Regeneration, College of Dentistry, University of Illinois at Chicago, Chicago, Illinois, United States of America; 3 Oregon Health and Science University, School of Dentistry, Portland, Oregon, United States of America; University of Tennessee, United States of America

## Abstract

Keratinocytes cover both the skin and some oral mucosa, but the morphology of each tissue and the behavior of the keratinocytes from these two sites are different. One significant dissimilarity between the two sites is the response to injury. Oral mucosal wounds heal faster and with less inflammation than equivalent cutaneous wounds. We hypothesized that oral and skin keratinocytes might have intrinsic differences at baseline as well as in the response to injury, and that such differences would be reflected in gene expression profiles.

## Introduction

Cutaneous wound healing is a multi-step process that nearly always ends with scar formation. The resultant scars range from those having little or no impact on physiologic function to hypertrophic scarring and contractures may interfere with the tissue function. One important aspect of wound healing is re-epithelialization, the restoration of epithelium by keratinocytes during the proliferative phase. Upon injury epithelial cells in the immediate vicinity of the wound edges undergo a proliferative and migratory burst and effectively replace those keratinocytes lost as a result of the injury [22]. Keratinocyte function is critical for effective wound re-epithelialization.

The healing of oral mucosal wounds proceeds through similar stages as that of skin wounds [37]. However, wound healing in the oral mucosa is clinically distinguished from skin healing in terms of both its rapidity and relatively minimal to no scar formation [39]. Studies in our laboratory have shown that in comparison to skin wounds, oral wounds exhibit a lower inflammatory response with lower neutrophil, macrophage, and T-cell infiltration [6], [33]. Similar to changes in inflammatory cytokines, oral and skin wounds also have differences in the expression of TGF-ß1, a pro-inflammatory, pro-fibrotic cytokine implicated in the etiology of hypertrophic scars [29]. The production of Vascular Endothelial Growth Factor (VEGF), a dominant mediator of wound angiogenesis, is significantly less in oral vs. skin wounds, and angiogenesis in oral wounds is less than in skin [7], [32].

Our laboratory has previously described the comprehensive and dynamic gene expression profile in skin and mucosal wounds throughout all the stages of wound healing. Using microarray technology, we have shown that although the expression patterns are similar in both tissues during healing, they are not identical [6]. Specifically, the genomic expression pattern of the injury response of oral mucosa is more rapid, shorter in duration, and of lesser intensity than the response of skin. These observations support the concept that oral wounds heal by kinetics differently from skin. One obvious explanation for this difference is the environmental variation of two sites, such as temperature, salivary flow, or microflora. However, studies have shown skin transposed into oral cavity maintains its morphologic characteristics [4], and transposed skin may result in an intraoral keloid [25]. These findings imply that the repair in oral mucosa is likely to involve intrinsic characteristics of mucosal tissue and is not simply due to environmental factors. Anatomic variation between oral mucosa and skin epithelium may also play a part in the healing differences noted between these two sites. Even though both oral mucosa and skin are stratified epithelium, structural differences between these two sites do exist. The presence of hair follicles and sweat glands occurs in skin but not in mucosa, while taste buds are found in mucosa but not in skin.

Given the significant differences in healing of wounds in skin and mucosa, intrinsic keratinocyte characteristics seem likely to be a differentiating factor. When excisional skin and oral mucosal wounds of equivalent size (1 mm) were compared, oral wounds exhibited rapid re-epithelialization with 100% closure at 24 hours post-injury. In contrast, cutaneous wounds were less than 25% re-epithelialized at a 24 hour time point [29]. This data suggests the proliferative capacity of oral keratinocytes is greater than that of skin keratinocytes. The differences observed in the tissue response to injury in mucosa and skin and the critical role keratinocytes play in these tissues suggests the possibility that the keratinocytes residing in different tissue sites might themselves be intrinsically different both at baseline and in response to trauma. Site specific differences related to anatomical position have also been documented in other cell types, such as fibroblasts and adipocytes [5], [11]. However to date, there are few reports describing differences in gene expression in keratinocytes from different locations. In current study we focus on identifying key inherent differences between oral and skin keratinocytes which mediate differential wound closure.

## Materials and Methods

### Ethics Statement

All animal procedures were approved by the University of Illinois Institutional Animal Care and Use Committee. Paired human skin (arm) and oral mucosal (hard palate) tissues (2 mmX10 mm) were obtained from healthy adult donors (18–35 years old) after consent under a protocol approved by Institutional Review Board at the University of Illinois at Chicago. Participants for this study signed written consent forms.

### Mouse keratinocytes for microarray analysis

Four (6–8 week old) female Balb/c mice (Harlan Inc., Indianapolis, IN) were sacrificed and skin epidermal tissues were obtained from the tail of mice, and oral epidermal tissues were obtained from the hard palate. Enzymatically isolated epithelium was used for analysis. Epithelium was separated from the dermis by 0.2% dispase treatment for 2 hrs at room temperature. All animal procedures were approved by the University of Illinois Institutional Animal Care and Use Committee.

### Total RNA preparation

Total RNA was extracted from the whole epithelial sheets using TriZol (Invitrogen, Carlsbad, CA) and purified by RNeasy kit (QIAGEN, Valencia, CA). The integrity (18S/28S) and concentrations of RNA was determined using an Experion (Bio-Rad, Hercules, CA) per the manufacturer's instruction.

### Microarray analysis

For genomic analyses, paired mRNA from mouse skin and palate (n = 4) epithelium was subjected to Affymetrix GeneChip Mouse Genome 430 v 2.0 Hybridizations x 8. The data was analyzed to identify genes whose expression differed between the two tissues. It was analyzed in Partek Genomics Suite statistical package. Hybridization signal intensities were normalized by quantiles and summarized using the Robust Multi-array Average. A False discovery rate (FDR) of <0.05 was used to identify significant positional differences in gene expression.

Ingenuity Pathways Analysis computes a score for each network according to the fit of the user's set of significant genes. The score is derived from a p-value and indicates the likelihood of the Focus Genes in a network being found together due to random chance. A score of 2 indicates there is a 1 in 100 chance that the Focus Genes are together in a network due to random chance. Therefore, scores of 2 or higher have at least a 99% confidence of not being generated by random chance alone. Biological functions are then calculated and assigned to each network.

Functional analysis of the data was done through the use of Ingenuity Pathways Analysis. The Functional Analysis identified the biological functions that were most significant to the data set. Molecules from the dataset that met the >10 fold cutoff of FDR <0.01 and were associated with biological functions in Ingenuity's Knowledge Base were considered for the analysis. Right-tailed Fisher's exact test was used to calculate a p-value determining the probability that each biological function assigned to that data set is due to chance alone. A heat map was generated by hierarchical clustering with squared Euclidean distance between the samples and complete linkage. The packages "hclust" and "heatmap" in R (http://www.r-project.org) were used. GEO Submission: GSE56135.

### Isolation of human primary skin and oral mucosal keratinocytes

Paired human skin (arm) and oral mucosal (hard palate) tissues (2 mmX10 mm) were obtained from healthy adult donors (18–35 years old) after consent under a protocol approved by Institutional Review Board at the University of Illinois at Chicago. The tissues were rinsed in 70% alcohol, washed with PBS containing 50 µg/mL gentamycin, and 0.5 µg/mL amphotericin B, and incubated for 2 hours at room temperature with 0.2% dispase solution. Separated epithelium was incubated for 10 min at 37°C in 0.05% trypsin and 0.53 mM EDTA (Invitrogen, Carlsbad, CA, USA) so a single cell suspension would be prepared. Following incubation, trypsin was neutralized with PBS containing 10 mg/mL Soybean Trypsin Inhibitor (Invitrogen, Carlsbad, CA, USA). The cells were suspended in KBM-2 and transferred into a 60-mm Petri dish at a density of 1 ×10^5^ cells *per* dish. Briefly skin and oral mucosal keratinocytes were cultured at 37°C and 5% CO_2_ in a humid atmosphere and grown in keratinocyte basal medium-2, KBM-2 (Cambrex, Walkersville, MD, USA). Experiments were performed at passage 2. For each assay: *in vitro* proliferation, gold migration and *in vitro* scratch wound closure, N = 3 paired human oral and skin keratinocytes were used. Triplet technical replicates were used for each N.

### *In Vitro* keratinocyte wound closure and proliferation assay

Keratinocytes were seeded in 6 and 12-well tissue culture plates for wound closure and proliferation assay. *In vitro* wounds were created by a scratch assay which involved the scraping of a 75–80% confluent keratinocyte monolayer by a 200 µl (yellow) pipette tip both horizontally and vertically across the plate, creating a grid form. 4×4 scratches were made. Six independent human keratinocyte cultures were used for wound closure. The defined areas were photographed at 0, 6 and 24 hours after wounding and the number of cells in the initial denuded area were counted. A different set of six independent human keratinocyte cultures of both skin and oral derived cells at time points of 0, 24, and 48 hours after scratch were examined with CellTiter96 Aqueous One Solution Cell Proliferation Assay (Promega, Madison, Wisconsin), which utilizes a colorimetric indicator to measure cell proliferation. Proliferation ratio was determined by the formula: OD_X hrs post-scratch_/OD_0 hrs post-scratch_. OD was read at an absorbance of 490 nm.

### *In Vitro* keratinocyte migration assay

The gold salt phagokinetic migration assay, which was first described by Albrecht-Buehler in 1977, has been routinely used to directly evaluate keratinocyte motility without the confounding possibility of cell proliferation. Tissue culture slides were dipped in 1% solution of bovine serum albumin and drained, then dipped in 100% ethanol, and rapidly dried. 1.8 ml HAuCl_4_ (14.5 mM) (Fisher Scientific, Pittsburg, PA) was added to 6 ml of 36.5 mM Na_2_CO_3_ (Sigma, St. Louis, MO) and diluted with 11 ml H_2_O. The solution was heated just to boiling and 1.8 ml of 0.1% formaldehyde was added. Two ml of the hot (80–90°C) gold particle suspension was added to culture slides. After 45 min the gold solution was aspirated, and slides re-coated for 1 hour at room temperature with fibronectin in HBSS (50 µg/ml). Freshly trypsinized subconfluent keratinocytes were seeded at a density of 2.5×10^3^ per slide. The cells were incubated for 18 hours at 37°C to allow adherence and migration. Afterwards, slides were washed gently to remove nonadherent cells and fixed in 0.1% formaldehyde in PBS. The phagocytic tracts were observed and photographed under dark field illumination. Under these conditions, the gold coat appeared as a finely-granulated light-gray background, and the particle-free areas appeared black. To assess cell migration, we used computer analysis to monitor microscopic images of the tracks made by cells and measured the area of tracks. Twenty non-overlapping fields from each slide were digitally imaged using AmScope acquisition software (American Scope) and analyzed using ScionImage software (Maryland). Since the migration tracks of the cells were visible as black empty spaces against the background of bright gold-salt particles, the colorization tool in the software was used to identify and measure the area of each track in each field. The sum of the track areas in the field was divided by the total area of the field and multiplied by 100 to yield the percentage of each field taken up by tracks. This percentage was called the migration index (MI).

### RT-PCR Analysis

Total RNA was isolated from adult skin and mucosal keratinocytes using Trizol Reagent (Invitrogen). RT PCR experiments were performed from at least three donors' paired skin and oral keratinocytes. One microgram of total RNA from each sample was subjected to first strand cDNA synthesis using M-MLV Reverse Transcriptase (Ambion) and human VEGF primers (Forward: 5′-TTT CTG CTG TCT TGG GTG CAT TGG-3′ and Reverse: 5′-ACC ACT TCG TGA TGA TTC TGC CCT-3′); human IL-15 primers (Forward: 5′-ACA GAA GCC AAC TGG GTG AAT GT -3′ and Reverse: 5′-CTT GCA TCT CCG GAC TCA AGT GAA-3′); and human AKT3 primers (Forward: 5′-TTG CTT TCA GGG CTC TTG AT-3′ and Reverse: 5′- CAT AAT TTC TTT TGC ATC ATC TGG-3′). PCR mixtures were loaded onto MicroAmp 96-well PCR reaction plates (Applied Biosystems). Real time PCR was performed with SYBR Green Mastermix in StepOnePlus Real Time PCR System (Applied Biosystems). The housekeeping gene that was used was ribosomal protein large P0 (RPLP0) based on the findings of (Minner and Poumay 2009) for selection of housekeeping genes for human epidermal keratinocytes. RPLP0 Forward primer sequence: ATCAACGGGTACAAACGAGTC; Reverse primer sequence: CAGATGGATCAGCCAAGAAGG.

To study keratinocyte response to stimuli, subconfluent cells were treated with IL-1β, IL-6 and DMOG for 24 hrs, total RNA was isolated, and standard real-time PCR was performed following the manufacturer's instructions (Applied Biosystems). Gene expression differences between mucosal and skin keratinocytes at baseline and upon treatments (IL 1β, IL 6 and DMOG) by real time RT-PCR were quantified using the ΔΔC_t_ method. Ribosomal protein large P0 (RPLP0) was used as a housekeeping gene, which has been reported by Minner and Poumay (Minner and Poumay, 2009) to be an appropriate housekeeping gene for normalizing gene expression. Ribosomal protein large P0 (RPLP0) was used as a housekeeping gene, as suggested by (Minner and Poumay 2009) for normalizing keratinocyte gene expression.

### Statistical analysis

GraphPad software (GraphPad Software, San Diego, CA) was used to analyze quantitative data. All data are expressed as mean ± the standard error of the mean (SEM). Two-way ANOVA was used to evaluate grouped data, and a Bonferroni's post-test was used to determine differences between groups. Comparisons were considered statistically significant when p<0.05. To compare skin and oral VEGF, IL-15 and AKT3 baseline expressions a Student's *t*-test was performed. *P*<0.05 was considered statistically significant.

## Results

### Significant differences in gene expression exist between normal (unwounded) oral and skin epithelium tissues

**Figure 1:**
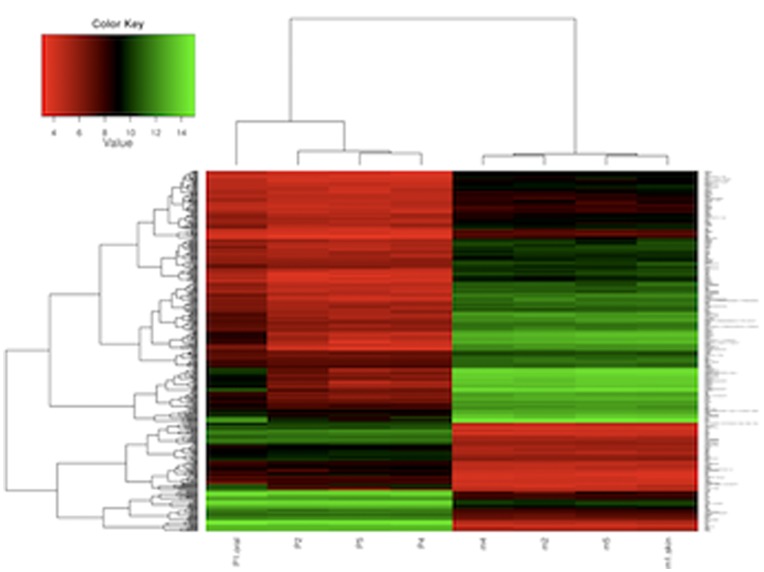
Hierarchical clustering of genes expressed more than A) 10-fold and B) 50-fold in oral and skin epithelium. The heatmap indicates gene expression in the skin and oral epithelium is significantly different. The color key shows the assignment of color to the expression intensity value. FDR<0.01, N = 4. The clustering is based on hierarchical clustering with squared Euclidean distance between the samples and complete linkage. The heatmaps were generated using package "gplots” in R (http://www.r-project.org).

We investigated differences in global gene expression between oral and skin freshly harvested epithelial sheets. Mouse epithelial tissues were chosen for analysis due to their availability and low variance between samples. Isolated keratinocyte sheets, independent of their underlying fibroblast layer were subjected to microarray analysis. A total of 13,710 genes were differentially expressed between oral and skin epithelial cells. Among the genes that were differentially expressed between skin and mucosa, 107 showed more than 10-fold expression in oral epithelium at a threshold of FDR<0.01. In contrast, 216 genes were more than 10-fold expressed in skin epithelium at the same threshold. A heatmap was generated to cluster genes expressed more than 10-fold and more than 50-fold (Fig. 1). This analysis demonstrates baseline epithelial gene expression is dramatically different between mucosa and skin, and that well defined clustering of gene expression is seen for each tissue type.

To appreciate the biological processes and molecular functions of the genes differentially expressed between oral and skin epithelium, we analyzed the dataset using the Ingenuity Pathway Analysis (Ingenuity Systems) [13]. Genes used as starting point for generating biological networks are referred to as focus genes in Ingenuity Pathways Analysis. Focus genes are then used to generate biological networks. The application is optimized to generate highly connected networks based on physical and functional interactions between focus genes and all other genes (and gene products) stored in the Ingenuity Pathways Knowledge Base (Table S1-S6 in File S1). Out of the 107 genes upregulated at least 10-fold in oral epithelium and 42 focus genes were identified. Of the 216 genes upregulated at least 10-fold in skin epithelium, 70 focus genes were used to generate top biological networks (Table S1-S6 in File S1).

**Figure 2:**
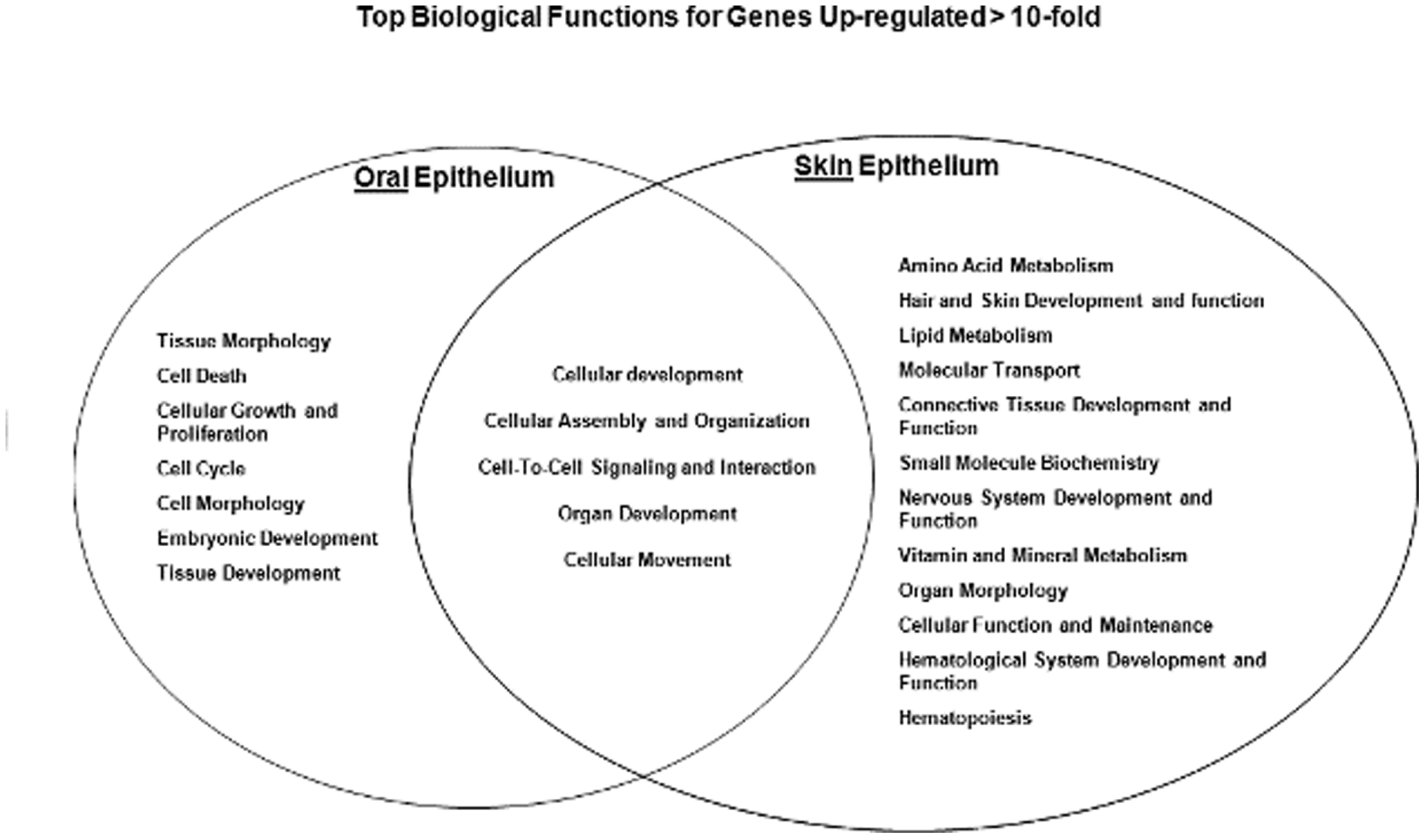
Top Biological Functions at each location. Fischer's exact test was used to calculate a p-value (<0.01) determining the probability each biological function assigned to data set is due to chance alone. Ingenuity Pathway Analysis software.

Using the pathways analysis, a diagram was generated to demonstrate the biological functions shared by both oral and skin epithelium, as well as those that are distinct to each location (Fig. 2A). Functions such as cellular development, cellular assembly and organization, cell-to-cell signaling and interaction, organ development, and cellular movement were shared by both oral and skin epithelium. Non-shared functions included hair and skin development, which was specific to skin, while the expression of cellular growth and proliferation was specific to oral epithelium. We then focused our analysis on genes related to keratinocyte proliferation and migration due to the critical role of these functions in wound closure. At a significance threshold of FDR<0.01, several proliferative and migration associated genes were expressed higher in oral epithelium in comparison to skin epithelium (Table 1). These findings implicate inherent tissue specific differences in proliferative and migratory capacity as contributors to the differential response to wound healing in skin and mucosa.

**Table 1:** Differentially Expressed Molecules Related to Cellular Movement and Proliferation.

Oral Epithelium		Skin Epithelium	
Molecules*	Fold Change	Molecules*	Fold Change
KRT13	1211	CD36	37
KRT4	637	ALCAM	20
PITX2	243	MMP3	14
SLPI	80	KLK6	8
AHSG	11		

*Keratin 13 (KRT13), Keratin 4 (KRT4), Paired-like homeodomain 2 (PITX2), Secretory Leukocyte Peptidase Inhibitor (SLPI), Alpha-2-HSglycoprotein (AHSG), CD36 molecule (thrombospond receptor) (CD36), Activated Leukocyte Cell Adhesion Molecule (ALCAM), Matrix Metallopeptidase 3 (MMP3), Kallikrein-related peptidase 6 (KLK6).

### Oral keratinocyte wound closure is faster than that of skin keratinocytes

**Figure 3:**
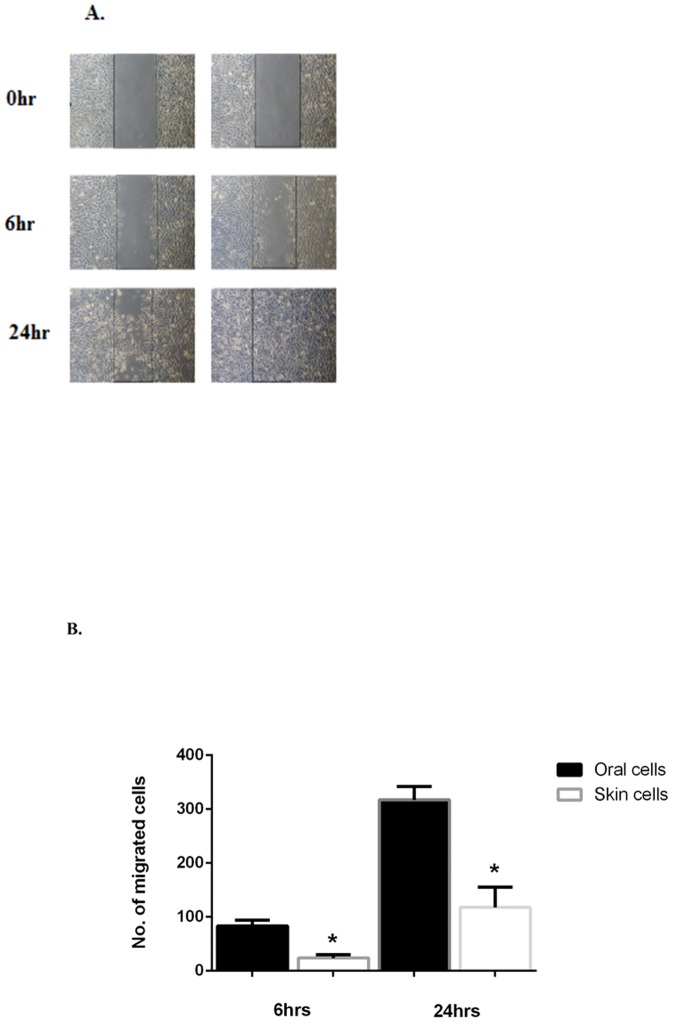
Oral and Skin keratinocyte wound closure after *In Vitro* wound scratching. Human primary oral and skin keratinocyte monolayers were wounded by scratches. **A**) The defined areas were photographed at 0, 6 and 24 hours after wounding. **B**) The number of cells in the denuded initial area were counted. Number of cells 6 hours post scratch: oral 83.4±10.9 vs skin 23.8±6.4. Number of cells 24 hours post scratch: oral 317.3±24.8 vs skin 118.0±37.6 *P<0.01. Student's t-test was used to compare the difference between oral and skin wounds at each time point and two-way ANOVA was used to evaluate grouped data over time. N = 3.

To investigate site-specific differences in epithelial migration and proliferation capacities, we performed functionality assays. Our goal was to understand the proliferative capability of oral keratinocytes independent of their environment and interactions with other cell types. In order to most faithfully reproduce the *in vivo* response, we studied primary keratinocytes from healthy, adult skin and palate tissues rather than established cell lines, since cell lines are often characterized by significant changes in growth regulation during immortalization. We utilized an *in vitro* wounding model in which a confluent keratinocyte monolayer was scratched with a pipette tip in a grid form and the number of cells that covered the initial denuded area were counted. After applying this model to both oral and skin keratinocytes we observed that wound closure was faster in oral keratinocytes in comparison to skin keratinocytes as early as 6 hours post scratch. The number of cells 6 hours post scratch in oral *in vitro* wounds was 83.4±10.9 vs skin 23.8±6.4. The number of cells 24 hours post scratch was: oral 317.3±24.8 vs skin 118.0±37.6 (Fig 3). Wound closure consists of keratinocyte proliferation and migration. We next dissected each function separately.

### Oral keratinocyte have intrinsic proliferation and migration capabilities that are higher than that of skin keratinocytes

**Figure 4:**
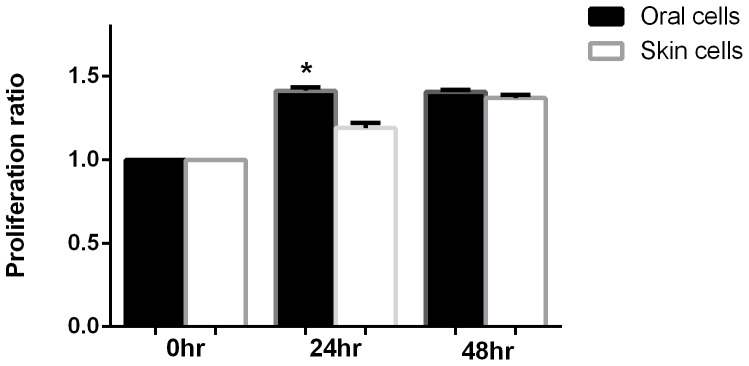
Comparison of skin and oral keratinocyte proliferation after *In Vitro* wounding. Isolated primary paired human keratinocytes (skin and hard palate) were cultured for *In Vitro* scratch assays. Keratinocyte proliferation was measured at time points of 0, 24, and 48 hours post scratch with CellTiter96 Aqueous One Solution Cell Proliferation Assay. Proliferation ratio was determined by the formula: OD_X hrs post-scratch_/OD_0 hrs post-scratch_. OD was read at an absorbance of 490 nm. 24 hours post scratch skin 1.190±0.03 vs oral 1.413± 0.02; 48 hours post scratch skin 1.370±0.02 vs oral 1.408±0.01 * p<0.05 by two-way ANOVA and Bonferroni post-test in skin vs oral wounds. N = 3. *Compared to 24 hr skin.

The proliferation of six independent keratinocyte cultures of both skin and oral derived cells was examined at time points of 0, 24, and 48 hours after scratch. Proliferation of oral keratinocytes was modestly but significantly faster than skin keratinocytes at 24 hour post scratch (skin 1.19±0.03 vs oral 1.41± 0.02) (Fig. 4). This data supports the concept oral keratinocytes maintain a higher proliferative capacity than skin keratinocytes.

**Figure 5:**
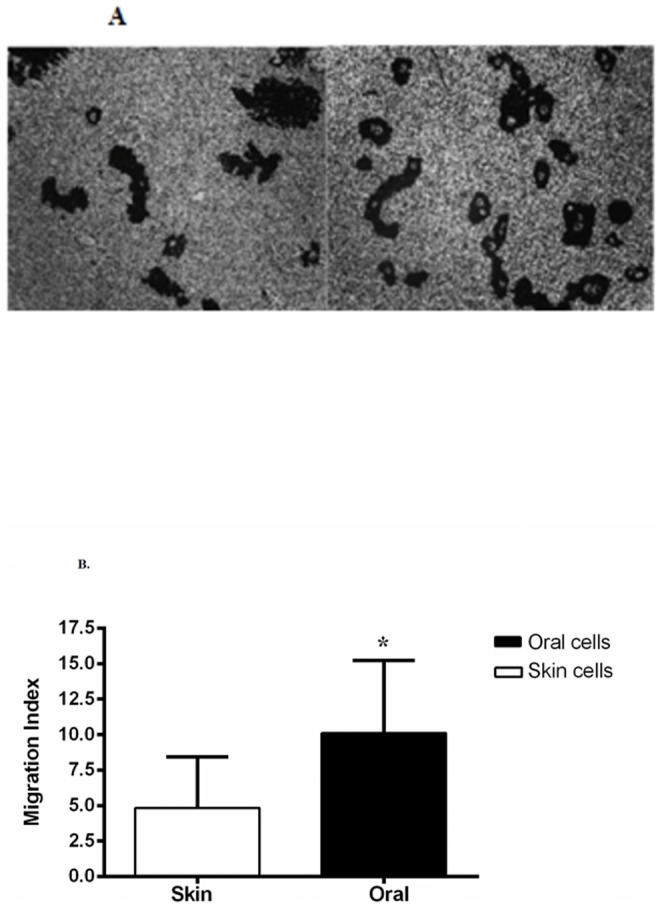
Comparison of skin and oral keratinocyte migration by Gold-Colloidal Migration Assay. Photographs are representative examples of the tracks made by single cells. The sum of the track areas in the field was divided by the total area of the field and multiplied by 100 to yield the percentage of each field taken up by tracks. This percentage was called the migration index (MI). **A**) Representative migration assay slides of paired human skin and oral keratinocytes. **B**) Skin keratinocyte MI 5.1±1.5 and oral keratinocyte MI 13.0±2.7 respectively. Average migration fold ratio of (2.6 fold) oral/skin of N = 3 independent paired oral and skin keratinocyte migration assays.

Since re-epithelialization includes both keratinocyte proliferation and migration, we next compared migration between oral and skin epithelium. To examine migration, we utilized a gold salt phagokinetic migration assay which has been routinely used to reliably and directly evaluate keratinocyte mobility without the confounding variable of cell proliferation. A representative paired human oral and skin keratinocyte migration assay is shown in Fig. 5A. Quantification of migration demonstrated that oral keratinocytes migrate at an average rate that is 2.6 fold faster than skin keratinocytes (Fig. 5B). Similar to the attribute of enhanced proliferation, the increased migratory capacity would be expected to facilitate wound closure in mucosa as compared to skin.

### Differential responses to stimuli between oral and skin keratinocytes

**Figure 6:**
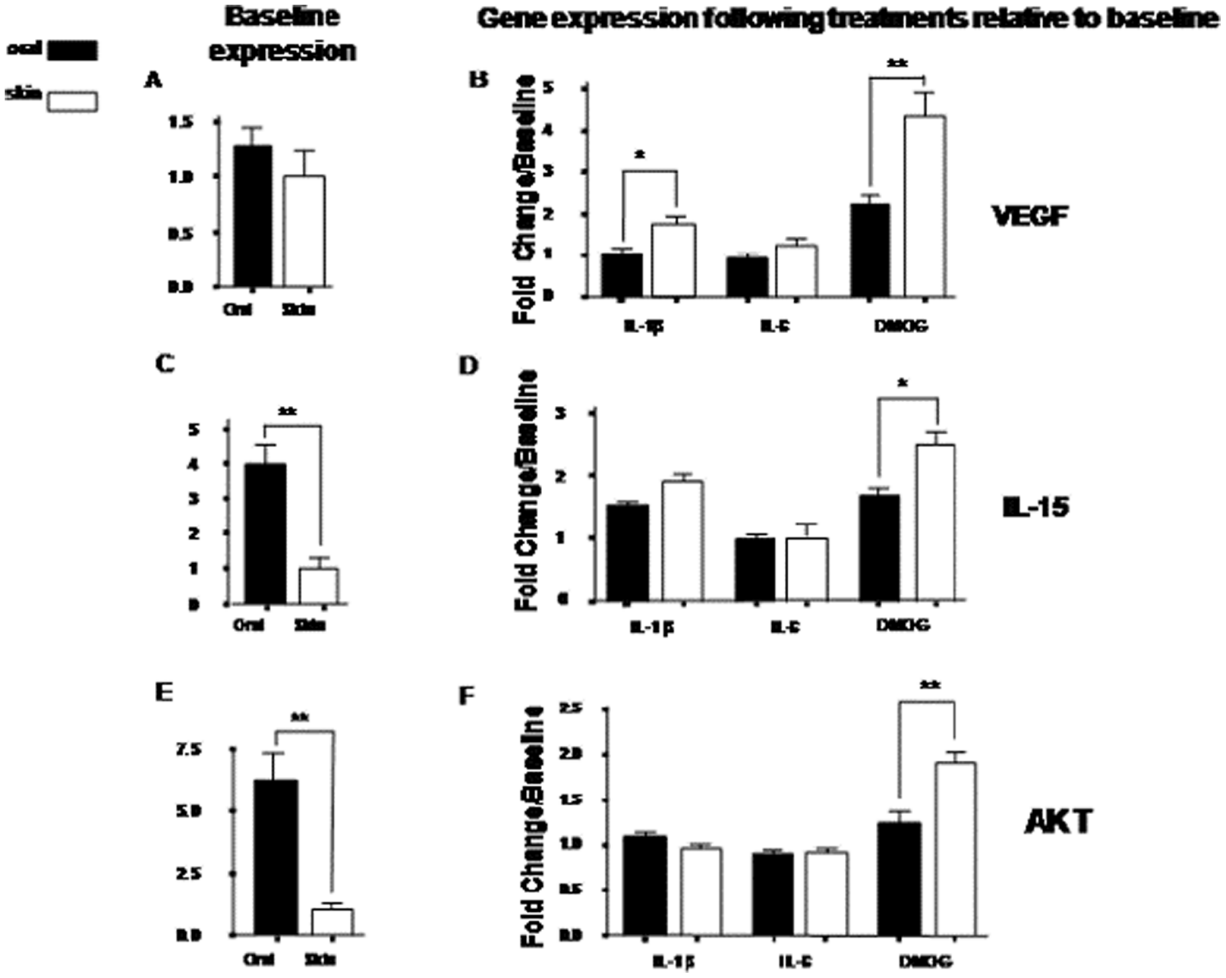
VEGF, IL-15, and AKT3 expression in skin and oral keratinocytes treated with IL-1β, IL-6, and DMOG (HIF 1α stimulant). Results were based on 3 individual donors' paired skin and oral mucosal keratinocytes. **A**) Baseline VEGF expression in oral and skin keratinocytes by RT PCR analyses. **B**) Relative VEGF expression in keratinocytes following stimulation. **C**) Baseline IL-15 expression in oral and skin keratinocytes by RT PCR analyses. **D**) Relative IL-15 expression in keratinocytes following stimulation. **E**) Baseline AKT3 expression in oral and skin keratinocytes. **F**) Relative AKT3 expression in keratinocytes following stimulation.

To test the hypothesis that oral and skin keratinocytes have differential response to injury, human primary cells from palate and skin were stimulated with a standardized dose of IL-1ß or IL-6, two cytokines known to be highly produced in the early wound. To mimic response to hypoxia, an important trigger in the early wound, keratinocytes were exposed to dimethyloxalyglycine (DMOG), an agent that augments hypoxia-inducible factor activity and mimics intracellular hypoxic conditions (Asikainen 2005). Following stimulation by cytokines or DMOG, levels of three sentinel factors, VEGF, IL-15 and AKT3, were evaluated by real time PCR (Figure 6). These factors were chosen because they are important components in many physiologic and pathologic processes such as wound healing and cancer [1], [10], [14], [16], [20], [24], [28], [34], [36], [40].

VEGF, a factor that plays a significant role in promoting and sustaining angiogenesis, is primarily produced by keratinocytes following wounding [32]. At baseline, VEGF expression levels in the oral keratinocytes were slightly, though not significantly, greater than that of the skin keratinocytes by RT-PCR analyses (Figure 6A). Following exposure to either IL1- ß or DMOG, VEGF expression was noted to be significantly upregulated in skin versus oral keratinocytes, (Figure 6B, 1.8 vs. 1.04 fold (p<0.05) in IL-Iβ treatment; 4.4 vs. 2.2 fold (p<0.01) in DMOG treatment). In contrast, changes in VEGF production after IL-6 treatment were similar in keratinocytes from skin and mucosa (Figure 6B).

IL-15, another factor that modulates wound healing, plays a critical role in the development and maturation of gamma delta intraepithelial T lymphocytes, a cell type known to play an important role in wound healing [14]. IL-15 is also a unique immuno-regulator in the resolution of excessive inflammation via induction of TGF- ß 1-producing cells [14]. RT-PCR showed that IL-15 expression in oral keratinocytes was significantly greater than that of skin keratinocytes at baseline (Figure 6C). Treatment of skin keratinocytes with either IL-1 ß or IL-6 showed no significantly greater IL-15 up-regulation than oral keratinocytes (Figure 6D). However following DMOG treatment, skin keratinocytes expressed more IL-15 than oral keratinocytes 2.5 vs. 1.7 fold (p<0.05) (Figure 6D).

AKT3 served as a final sentinel gene that we examined in the injury response of oral and skin keratinocytes. AKT (a serine/threonine protein kinase) enzymes regulate multiple cellular processes including cell survival, cell cycle, cell proliferation, cell migration, and glycogen and protein metabolism [28]. Akt3 was selected as a sentinel for evaluation due to the above roles, which are important in wound healing and cancer. Baseline AKT3 expression in oral keratinocytes was significantly greater than that of the skin keratinocytes by RT-PCR (Figure 6E). Treatment of oral mucosal or skin keratinocytes with IL-1 ß or IL-6 resulted in almost no change in the expression of AKT3 in either population (Figure 6F). However, treatment with DMOG led to significantly more up-regulation of AKT3 in skin versus oral keratinocytes (1.9 vs. 1.2 fold, p<0.001, Figure 6F).

In summary, at baseline the expression level of VEGF in the oral keratinocytes was slightly greater than that of the skin keratinocytes; the baseline expression levels of IL-15 and AKT3 in the oral keratinocytes were greater than that of the skin keratinocytes. However, under stimulation conditions, especially under hypoxic conditions (DMOG treatment), skin keratinocytes showed significantly higher expression of VEGF, IL-15 and AKT3 than oral keratinocytes.

## Discussion

Studies in at least three different models (human, pig, and mouse) demonstrate that oral wound healing occurs faster and with less scarring than skin wound healing [6], [21], [33]. Intrinsic differences in growth factor production, stem cell levels, and cellular proliferation capacity have all been suggested to support oral mucosal wound repair [2], as well as environmental influences, including saliva, have also been suggested to play a role [3]. The current findings suggest that the superior repair in oral mucosa is supported by intrinsic characteristics of the epithelium.

To assess site-specific differences in oral and skin keratinocyte function, we first examined the baseline gene expression profiles of these two tissues in comparison to each other. The observation that 13, 710 genes are differentially expressed between the two tissues was unexpected, as the tissues share many histologic and functional characteristics. This finding that gene expression profiles of oral and skin epithelium are quite different suggests that such dissimilarities may be critical to function. From our analysis, we observed differential expression of specific proliferation and migration associated genes in oral and skin epithelium (summarized in Figure 3). Several differentially expressed genes might influence keratinocyte migration and proliferation. For example, the expression of Pitx2, a protein know to support migration and proliferation, was noted to be enhanced in oral mucosal keratinocytes [15]. Several genes noted to play a role in cell migration were also differentially expressed between the two tissues, with some higher in oral and some higher in skin keratinocytes (Fig 3). AHSG, which was expressed at high levels by oral keratinocytes, has been reported to increase the migration of keratinocytes [38]. In contrast, CD36, a molecule known to increase the migration of melanoma cells treated with laminin and fibronectin, was expressed at higher levels in skin than mucosa [35]. Both ALCAM and KLK6 proteins increase cellular migration and were more highly expressed in skin. MMP 3 protein, also more highly expressed in skin, is relevant to the migration of HaCaT cells [9]. Together, this data demonstrates the constellation of proteins that regulate migratory capacity is quite different in keratinocytes of skin and mucosa.

Fibroblasts from different anatomical sites are known to maintain intrinsic differences [27]. As compared to skin, oral mucosal fibroblasts exhibit enhanced proliferative capacity and an altered contractility profile [30]. Skin released steroids seems to be cell type-dependent and under regulation by external factors such as ultraviolet radiation. They behave in intracrine, autocrine or paracrine pathways to regulate local homeostasis [31]. Both fibroblasts and epithelial cells appear to maintain positional identities that contribute to the superior repair of oral mucosa. Differential epithelial responses are very likely to be important to the site-specific differences in repair of mouth and skin wounds.

The current study clearly shows, when compared to skin, isolated oral keratinocytes exhibit accelerated migration and greater proliferation. Keratinocytes independent of their underlying fibroblast influence have not been studied previously. One caveat that we did not specifically consider is the interaction of fibroblasts and epithelial cells, an interaction that may also be important to site specific healing outcomes. Several studies suggest fibroblasts might influence epithelial cells, and vice versa, and such epithelial-connective tissue interactions are important to wound healing outcomes [23]. *In vitro* co-culture experiments, signals from overlying keratinocytes influence collagen synthesis and the activity of underlying fibroblasts [8].

While prior studies have reported that oral mucosa has a higher proliferative rate than skin [12], our findings are the first to compare human oral and skin keratinocyte proliferation independent of their underlying connective tissue and environment. Our data suggests that oral epithelial cells have an intrinsic proliferative capacity greater than skin that is independent of modulation by the underlying connective tissue. We demonstrate that oral keratinocytes migrate 2.6 times faster than skin keratinocytes in paired human primary cultures independent of underlying fibroblasts. While fibroblasts may be a source of critical paracrine motogenic factors, such as KGF-1 and 2, and dermal matrix metalloproteases (MMPs) in wounds, our studies eliminate this effect. Faster migration exhibited by oral keratinocytes therefore seems to be intrinsic. The differences between the migratory capacity of oral and skin keratinocytes could involve several factors. Differential expression of motogenic autocrine growth factors, matrix metalloproteases and integrins may occur between oral and skin keratinocytes. For example, MMP 9 is a type IV collagenase that is expressed by migrating but not quiescent skin keratinocytes. A transient upregulation of MMP-9 is observed in the migrating keratinocytes of acute skin wounds, with levels decreasing as re-epithelialization is complete [19]. In contrast to the epidermis, MMP-9 is expressed in oral mucosal keratinocytes even in the quiescent state [26]. MMP-9 expression is also detected in the migrating epithelial outgrowth of mucosal wounds [26]. This type of baseline intrinsic difference might contribute to an accelerated migratory capacity in oral epithelium. The differential migratory activity of oral and skin keratinocytes might derive from their differential patterns of interaction with extracellular matrix proteins. Our experiments compared migration on fibronectin, which is one of the most critical extracellular matrix proteins upon which keratinocytes migrate *in vivo*. Different oral and skin keratinocytes may express different complements of integrins that influence cell migration on fibronectin. Migrating keratinocytes are also believed to produce their own extracellular matrix molecules to support their migration; such functional protein production may also be different in oral and skin keratinocytes.

By examining three sentinel genes, the studies here provide specific information to show that oral and skin keratinocytes respond differently to stimuli commonly found in the wound environment, including IL-1 ß IL-6, and hypoxia. After IL-1 ß and DMOG treatment, expression of VEGF and IL-15 showed greater up-regulation in skin than in oral keratinocytes. Our results are consistent with a previous report which showed greater VEGF production in skin wounds than in oral wounds [32]. Similarly, DMOG treatment demonstrates a greater up-regulated AKT3 expression in skin keratinocytes compared to oral keratinocytes as well. Differential responses of oral and skin keratinocytes to stimulation have been reported before [16], [17], [18]; our results further demonstrate this differential response profile. IL-6 had no significant effect on the VEGF, IL-15 and AKT3 regulation in both oral and skin keratinocytes. This suggests that VEGF, lL-15 or AKT3 production may not be dependent on IL-6 stimulation alone or that the keratinocyte response to IL-6 may require other factors. Chen *et al.* (2012) show that mucosal wounds heal under conditions of significantly less hypoxia than skin wounds. Some of this difference may derive from baseline differences, since the normal tongue has a higher baseline vascularity than does skin [7]. In addition, cellular responses to hypoxia are mediated by hypoxia-inducible factor (HIF)-1 signaling and skin wounds have a higher levels of HIF-1α in comparison to mucosal wounds [7]. Decreased hypoxia may also explain why the expression of VEGF, a factor downstream of HIF-1α, is reduced in oral versus skin wounds [32]. The overall pattern suggests that skin keratinocytes exhibit a more robust response to injury than oral keratinocytes. This may be the result of oral keratinocytes having a more advantageous gene expression profile at baseline which allows for a more rapid or controlled response to injury.

Additional studies will be needed to identify the critical differences that lead to enhanced proliferation and migration seen in oral mucosal keratinocytes when compared to those from skin. In the future, the detailed examination of genetic elements and factors that are important for the accelerated proliferation and migration of oral keratinocytes might suggest candidate molecules that could be used to enhance skin wound healing.

## Supporting Information

File S1 **Table S1**. * The network Score is based on the hypergeometric distribution and is calculated with the right-tailed Fisher's Exact Test. The score is the –log (Fisher's Exact test result). Networks are scored for the likelihood of finding the focus molecule(s) in that given network. The higher the score, the lower the probability that one would find the focus molecules(s) in a given network by random chance. ** Networks are preferentially enriched for focus molecules (shown in bold) with the most extensive interactions, and for which interactions are specific with the other molecules in the network (rather than molecules that are promiscuous—those that interact with a broad selection of molecules throughout Ingenuity's knowledge base). Additional non-focus molecules from the dataset and from Ingenuity's knowledge base are then recruited and added to the growing networks. **Table S2**. * The network Score is based on the hypergeometric distribution and is calculated with the right-tailed Fisher's Exact Test. The score is the –log (Fisher's Exact test result). Networks are scored for the likelihood of finding the focus molecule(s) in that given network. The higher the score, the lower the probability that one would find the focus molecules(s) in a given network by random chance. ** Networks are preferentially enriched for focus molecules (shown in bold) with the most extensive interactions, and for which interactions are specific with the other molecules in the network (rather than molecules that are promiscuous—those that interact with a broad selection of molecules throughout Ingenuity's knowledge base). Additional non-focus molecules from the dataset and from Ingenuity's knowledge base are then recruited and added to the growing networks. **Table S3**. * Fischer's exact test was used to calculate a p-value determining the probability that each biological function assigned to that data set is due to chance alone. **Table S4**. * Fischer's exact test was used to calculate a p-value determining the probability that each biological function assigned to that data set is due to chance alone. **Table S5**. *Fischer's exact test was used to calculate a p-value determining the probability that each biological function assigned to that data set is due to chance alone. **Table S6**. *Fischer's exact test was used to calculate a p-value determining the probability that each biological function assigned to that data set is due to chance alone.
(DOC)
