# Supplementary material for: Intrinsic Differences between Oral and Skin Keratinocytes
Source: PLoS One. 2014 Sep 8;9(9):e101480. doi: 10.1371/journal.pone.0101480 (PMC4157746; doi:10.1371/journal.pone.0101480)
Supplement: File S1 — Table S1. * The network Score is based on the hypergeometric distribution and is calculated with the right-tailed Fisher's Exact Test. The score is the –log (Fisher's Exact test result). Networks are scored for the likelihood of finding the focus molecule(s) in that given network. The higher the score, the lower the probability that one would find the focus molecules(s) in a given network by random chance. ** Networks are preferentially enriched for focus molecules (shown in bold) with the most extensive interactions, and for which interactions are specific with the other molecules in the network (rather than molecules that are promiscuous—those that interact with a broad selection of molecules throughout Ingenuity's knowledge base). Additional non-focus molecules from the dataset and from Ingenuity's knowledge base are then recruited and added to the growing networks. Table S2. * The network Score is based on the hypergeometric distribution and is calculated with the right-tailed Fisher's Exact Test. The score is the –log (Fisher's Exact test result). Networks are scored for the likelihood of finding the focus molecule(s) in that given network. The higher the score, the lower the probability that one would find the focus molecules(s) in a given network by random chance. ** Networks are preferentially enriched for focus molecules (shown in bold) with the most extensive interactions, and for which interactions are specific with the other molecules in the network (rather than molecules that are promiscuous—those that interact with a broad selection of molecules throughout Ingenuity's knowledge base). Additional non-focus molecules from the dataset and from Ingenuity's knowledge base are then recruited and added to the growing networks. Table S3. * Fischer's exact test was used to calculate a p-value determining the probability that each biological function assigned to that data set is due to chance alone. Table S4. * Fischer's exact test was used to calculate a p-valu [file pone.0101480.s001.doc]

**Suplemental Data**

**Table S1.**

**Ingenuity Pathways Analysis: Top Networks—Associated Network Functions for Genes Upregulated more than 10-fold in Oral Epithelium**

**Top Functions** **Score*** **Focus** **Molecules**** **Molecules**

| Cellular Development, Organ Development, Tissue Development | 26 | 13 | **ADA**, **AHSG**, ALOX15, **ARG1**, **CALCRL**, cyclic AMP, DLX2, EGR1, FGF10, **FMO1**, Focal adhesion kinase, GCM1, GH1, **GPX2**, **GSTA4**, IL6, **ISL1**, NANOG, NEUROD1, NFE2L2, **NMU**, NR5A1, **PALLD**, **PITX1**, **PITX2**, POMC, POU1F1, PRL, **SOX2**, STAT3, TCF3, TNF, TWIST1, VEGFC |
| --- | --- | --- | --- |
| Gene Expression, Cellular Growth and Proliferation, Nervous System Development and Function | 25 | 13 | **BDNF**, beta-estradiol, **CADPS2**, **CASP1**, Ck2, COL1A1, **CRISP3 (includes EG:10321), CRYAB**, CSF1R, **DCN**, **DEFB4 (includes EG:56519),** dopamine, EGR1, ERBB4, **FMO2**, GNRH1, HMGB1 (includes EG:3146), Hsp90, IGF1, IL1, **IL1F6**, IRS1, L-glutamic acid, NCOA1, NFkB (complex), NRG1, **ODZ3**, **PLAGL1**, PML, POMC, SIN3A, **SLPI**, SUMO1, **TMEFF2**, TP53 |
| Gene Expression, Organ Development, Cellular Function and Maintenance | 25 | 13 | AHR, amino acids, APP, CDX1, CREB1, **CSRP2**, CTNNB1, CYP19A1, **CYP2A13**, **DSC2**, EGR1, **EYA1, EYA4, FETUB**, FGF3, HNF4A, **HS3ST1**, ID2, **IQGAP2, MEIS2**, NR5A1, **PITX2**, PML, POMC, retinoic acid, **RYR3**, **SIM2**, SMAD2, SMAD3, SMAD4, SUMO1, TCF3, TRIM24, **WNK4** |
| Reproductive System Development and Function, Hair and Skin Development and Function, Organ Development | 4 | 3 | beta-estradiol, CYP19A1, Cytokeratin, dopamine, EGF, EGFR, ESR1, GNRH1, KRT1, KRT2, **KRT4**, KRT5, KRT7, KRT9, KRT10, KRT12, **KRT13**, KRT14, KRT15, KRT16, KRT17, KRT19, KRT20, KRT23, KRT6A, Mapk, MAPK1, NCOA1, NCOA2, NCOA3, **PGR**, progesterone, SRC, TRIM24 |

* The network Score is based on the hypergeometric distribution and is calculated with the right-tailed Fisher's Exact Test. The score is the –log (Fisher's Exact test result). Networks are scored for the likelihood of finding the focus molecule(s) in that given network. The higher the score, the lower the probability that one would find the focus molecules(s) in a given network by random chance.

** Networks are preferentially enriched for focus molecules (shown in bold) with the most extensive interactions, and for which interactions are specific with the other molecules in the network (rather than molecules that are promiscuous--those that interact with a broad selection of molecules throughout Ingenuity's knowledge base). Additional non-focus molecules from the dataset and from Ingenuity's knowledge base are then recruited and added to the growing networks**.**

**Table S2.**

**Ingenuity Pathways Analysis: Top Networks—Associated Network Functions for Genes Upregulated more than 10-fold in Skin Epithelium**

**Top Functions Score* Focus Molecules** Molecules**

| Hematological System Development and Function, Cellular Development, Cellular Growth and Proliferation | 27 | 15 | **ADH1C (includes EG:126), ALCAM**, Calmodulin, CYTH3, Cytokeratin, dihydrotestosterone, EGF, EPO, GATA1, GATA2, **HOXA9, HOXC13, HOXD8, HOXD9, HOXD10, HSD3B1**, JUN, **KRT2**, KRT5, KRT7, **KRT33A, KRTAP6-3 (includes EG:337968)**, MEIS1, NFATC2, NFE2**, PADI3**, PBX1, PHF1, PKNOX1, **RGN, S100A3**, SIM1, **THBS1**, VAV1, VIP |
| --- | --- | --- | --- |
| Cellular Development, Developmental Disorder, Cardiovascular System Development and Function | 22 | 13 | **ASPN**, CDH1, COL2A1, Collagen(s), CSF1, CSF1R, **DCT**, **DDR2, FBP1**, IL1B, KIT, KLF10, LIF, MITF, MITF-p300/CBP, **MMP3**, MMP13, MMP14, **ORM2**, PVR, PVRL1, **PVRL3**, REN, RUNX1, **SAA2**, **SCD3, SILV, SLC39A8, STFA3**, testosterone, TFE3, TGFB1, TGFB2, TGFB3, **THBS1** |
| Lipid Metabolism, Small Molecule Biochemistry, Molecular Transport | 20 | 12 | ACLY, Ap1, **CD36**, **CD200**, **CXCL12**, DDX20, **EGR2, EGR3**, **FABP4**, Gata, **GATA3**, HDL, IL1RL1, Jnk, linolenic acid, LIPE, **LPL**, LTBP1, Mek, **MMP3**, MMP17, NCOR-LXR-Oxysterol-RXR-9 cis RA, NFkB (complex), octanoic acid, P38 MAPK, PVR, Rxr, **SCD**, STAT1/3/5 dimer, Tgf beta, TH2 CYTOKINE, **THBS1**, Timp, **TIMP3**, TLR6 |
| Cellular Assembly and Organization, Organismal Development, Lipid Metabolism | 17 | 11 | ADIPOQ, AGT, AHR, aldosterone, beta-estradiol, CDH1, CLDN6, EPO, ESR2, FASN, **FXYD4**, **GJA3, GPR37**, HGF, HSPA5, IGF1R, **KRT35**, LPIN1, MED1, **MGLL**, nitric oxide, NR1H3, PLCG1, PPARG, **RDH16, SNAP91, SPRR2D, SPRR2G (includes EG:6706), SUSD2**, TJP1, TLR2, TYR, **TYRP1**, Vegf, VIP |
| Gene Expression, Lipid Metabolism, Molecular Transport | 15 | 10 | ADIPOQ, BMP2, BMPR2, **CA6**, CAR ligand-CAR-Retinoic acid-RXRα**, COL4A1**, CSF1R, **CYP1B1**, Cyp2b, **CYP2B6**, **CYP2B19, CYP2G1P**, DCN, DDIT3, EPO, ERBB4, ethanol, FASN, fluoride, HAMP, IL10, LDL, **LRAT**, Ncoa-Nr1i2-Rxra, Ncoa-Nr1i3-Rxra, NOS2, NR1I2, **PDZRN3**, PXR ligand-PXR-Retinoic acid-RXRα, retinoic acid, Rxr, RXRA, **SLC40A1, TCHH**, Unspecific monooxygenase |
| Nervous System Development and Function, Tissue Morphology, Cellular Movement | 14 | 9 | ADCYAP1, ATN1, **ATP1B1**, BCL6, **CCDC80**, CNP, **CRYM**, DLG4, EBF1, **ELOVL3**, ERBB2, ERBB4, FYN, **GGCT**, HBEGF, HTT, IGFBP5, KRT7, **KRT27**, MPZ, NAB2, norepinephrine, NR1H3, NRP1, PMP22, **POU3F1**, progesterone, PTK2B, RGS16, SEMA3A, **SEMA3D**, SEMA3E, SMAD7, **TM4SF1**, Vegf |

* The network Score is based on the hypergeometric distribution and is calculated with the right-tailed Fisher's Exact Test. The score is the –log (Fisher's Exact test result). Networks are scored for the likelihood of finding the focus molecule(s) in that given network. The higher the score, the lower the probability that one would find the focus molecules(s) in a given network by random chance.

** Networks are preferentially enriched for focus molecules (shown in bold) with the most extensive interactions, and for which interactions are specific with the other molecules in the network (rather than molecules that are promiscuous--those that interact with a broad selection of molecules throughout Ingenuity's knowledge base). Additional non-focus molecules from the dataset and from Ingenuity's knowledge base are then recruited and added to the growing networks.

**Table S3.**

**Ingenuity Pathways Analysis: Top Bio Functions - Molecular and Cellular Functions for Genes Upregulated more than 10-fold in Oral Epithelium**

**Category P-value* # Molecules Molecules**

| Gene Expression | 7.49E-05 - 3.75E-02 | 13 | [ADA](https://analysis.ingenuity.com/pa/nodeview/nodeview.jsp?nodeid=ING%3A9lo), [BDNF](https://analysis.ingenuity.com/pa/nodeview/nodeview.jsp?nodeid=ING%3A3l0r), [CYP2A13](https://analysis.ingenuity.com/pa/nodeview/nodeview.jsp?nodeid=ING%3Aa9w), [DCN](https://analysis.ingenuity.com/pa/nodeview/nodeview.jsp?nodeid=ING%3Ab2e), [EYA1](https://analysis.ingenuity.com/pa/nodeview/nodeview.jsp?nodeid=ING%3A9kq), [ISL1](https://analysis.ingenuity.com/pa/nodeview/nodeview.jsp?nodeid=ING%3A639), [MEIS2](https://analysis.ingenuity.com/pa/nodeview/nodeview.jsp?nodeid=ING%3A5bb), [PGR](https://analysis.ingenuity.com/pa/nodeview/nodeview.jsp?nodeid=ING%3A8hw), [PITX1](https://analysis.ingenuity.com/pa/nodeview/nodeview.jsp?nodeid=ING%3Aba0), [PITX2](https://analysis.ingenuity.com/pa/nodeview/nodeview.jsp?nodeid=ING%3A94t), [PLAGL1](https://analysis.ingenuity.com/pa/nodeview/nodeview.jsp?nodeid=ING%3Aakv), [SIM2](https://analysis.ingenuity.com/pa/nodeview/nodeview.jsp?nodeid=ING%3A6ci), [SOX2](https://analysis.ingenuity.com/pa/nodeview/nodeview.jsp?nodeid=ING%3A5ie) |
| --- | --- | --- | --- |
| Cell Morphology | 1.25E-04 - 3.16E-02 | 6 | [ADA](https://analysis.ingenuity.com/pa/nodeview/nodeview.jsp?nodeid=ING%3A9lo), [ARG1](https://analysis.ingenuity.com/pa/nodeview/nodeview.jsp?nodeid=ING%3A8ol), [BDNF](https://analysis.ingenuity.com/pa/nodeview/nodeview.jsp?nodeid=ING%3A3l0r), [ISL1](https://analysis.ingenuity.com/pa/nodeview/nodeview.jsp?nodeid=ING%3A639), [PALLD](https://analysis.ingenuity.com/pa/nodeview/nodeview.jsp?nodeid=ING%3A18agn), [PITX2](https://analysis.ingenuity.com/pa/nodeview/nodeview.jsp?nodeid=ING%3A94t) |
| Cellular Development | 2.60E-04 - 3.85E-02 | 16 | [ADA](https://analysis.ingenuity.com/pa/nodeview/nodeview.jsp?nodeid=ING%3A9lo), [AHSG](https://analysis.ingenuity.com/pa/nodeview/nodeview.jsp?nodeid=ING%3A689), [ARG1](https://analysis.ingenuity.com/pa/nodeview/nodeview.jsp?nodeid=ING%3A8ol), [BDNF](https://analysis.ingenuity.com/pa/nodeview/nodeview.jsp?nodeid=ING%3A3l0r), [CADPS2](https://analysis.ingenuity.com/pa/nodeview/nodeview.jsp?nodeid=ING%3A42zx), [CASP1](https://analysis.ingenuity.com/pa/nodeview/nodeview.jsp?nodeid=ING%3A5c2), [CRYAB](https://analysis.ingenuity.com/pa/nodeview/nodeview.jsp?nodeid=ING%3A85v), [DCN](https://analysis.ingenuity.com/pa/nodeview/nodeview.jsp?nodeid=ING%3Ab2e), [EYA1](https://analysis.ingenuity.com/pa/nodeview/nodeview.jsp?nodeid=ING%3A9kq), [ISL1](https://analysis.ingenuity.com/pa/nodeview/nodeview.jsp?nodeid=ING%3A639), [KRT4](https://analysis.ingenuity.com/pa/nodeview/nodeview.jsp?nodeid=ING%3A916), [PALLD](https://analysis.ingenuity.com/pa/nodeview/nodeview.jsp?nodeid=ING%3A18agn), [PGR](https://analysis.ingenuity.com/pa/nodeview/nodeview.jsp?nodeid=ING%3A8hw), [PITX1](https://analysis.ingenuity.com/pa/nodeview/nodeview.jsp?nodeid=ING%3Aba0), [PITX2](https://analysis.ingenuity.com/pa/nodeview/nodeview.jsp?nodeid=ING%3A94t), [SOX2](https://analysis.ingenuity.com/pa/nodeview/nodeview.jsp?nodeid=ING%3A5ie) |
| Cell Death | 8.41E-04 - 3.85E-02 | 9 | [ADA](https://analysis.ingenuity.com/pa/nodeview/nodeview.jsp?nodeid=ING%3A9lo), [BDNF](https://analysis.ingenuity.com/pa/nodeview/nodeview.jsp?nodeid=ING%3A3l0r), [CADPS2](https://analysis.ingenuity.com/pa/nodeview/nodeview.jsp?nodeid=ING%3A42zx), , [CASP1](https://analysis.ingenuity.com/pa/nodeview/nodeview.jsp?nodeid=ING%3A5c2), [CRYAB](https://analysis.ingenuity.com/pa/nodeview/nodeview.jsp?nodeid=ING%3A85v), [DCN](https://analysis.ingenuity.com/pa/nodeview/nodeview.jsp?nodeid=ING%3Ab2e), [EYA1](https://analysis.ingenuity.com/pa/nodeview/nodeview.jsp?nodeid=ING%3A9kq), [ISL1](https://analysis.ingenuity.com/pa/nodeview/nodeview.jsp?nodeid=ING%3A639), PALLD |
| Cellular Growth and Proliferation | 9.10E-04 - 3.18E-02 | 15 | [ADA](https://analysis.ingenuity.com/pa/nodeview/nodeview.jsp?nodeid=ING%3A9lo), [AHSG](https://analysis.ingenuity.com/pa/nodeview/nodeview.jsp?nodeid=ING%3A689) ,[BDNF](https://analysis.ingenuity.com/pa/nodeview/nodeview.jsp?nodeid=ING%3A3l0r), CALCRL, CASP1, CRYAB, DCN, ISL1, KRT4, PGR, PITX2, PLAGL1, SLPI, SOX2, TMEFF2 |

***** Fischer's exact test was used to calculate a p-value determining the probability that each biological function assigned to that data set is due to chance alone.

**Table S4.**

**Ingenuity Pathways Analysis: Top Bio Functions - Molecular and Cellular Functions for Genes Upregulated more than 10-fold in Skin Epithelium**

**Category P-value* # Molecules Molecules**

| Cellular Development | 1.14E-05 - 4.93E-02 | 15 | Wdnm1-like, ATP1B1, CD200, CXCL12, EGR2, EGR3, FABP4, GATA3, HOXA9, HOXD11, MMP3, PU3F1, SCD, THBS1, TIMP3 |
| --- | --- | --- | --- |
| Amino Acid Metabolism | 3.14E-05 - 3.31E-02 | 4 | DCT, HAL, SILV, TYRP1 |
| Lipid Metabolism | 3.14E-05 - 4.93E-02 | 18 | ADH1C, CD36, CXCL12, CYP1B1, CYP2B19, CYP2G1P, ELOVL3, FABP4, FAR2, LPL, LRAT, MGLL, MMP3, RDH16, SAA2, SCD, SLC22A4, THBS1 |
| Molecular Transport | 3.14E-05 - 4.93E-02 | 13 | ADH1C, CD36, CXCL12, ELOVL3, FABP4, LPL, LRAT, MMP3, RDH16, SAA2, SCD, SLC22A4, THBS1 |
| Small Molecule Biochemistry | 3.14E-05 - 4.93E-02 | 23 | ADH1C, CD36, CXCL12, CYP1B1, CYP2B19, CYP2G1P, DCT, ELOVL3, FABP4, FAR2, GATA3, HAL, LPL, LRAT, MGLL, MMP3, RDH16, SAA2, SCD, SILV, THBS1, TYRP1, SLC22A4 |

***** Fischer's exact test was used to calculate a p-value determining the probability that each biological function assigned to that data set is due to chance alone.

**Table S5.**

**Ingenuity Pathways Analysis: Top Bio Functions - Physiological System Development and Function for Genes Upregulated more than 10-fold in Oral Epithelium**

**Category**  P-value* # Molecules Molecules

| Tissue Morphology | 5.13E-06 - 3.16E-02 | 11 | [ADA](https://analysis.ingenuity.com/pa/nodeview/nodeview.jsp?nodeid=ING%3A9lo), [ARG1](https://analysis.ingenuity.com/pa/nodeview/nodeview.jsp?nodeid=ING%3A8ol), [BDNF](https://analysis.ingenuity.com/pa/nodeview/nodeview.jsp?nodeid=ING%3A3l0r), CALCRL, DCN, IL1F6, PGR, PITX1, PITX2, RYR3, SIM2 |
| --- | --- | --- | --- |
| Embryonic Development | 1.25E-05 - 3.47E-02 | 11 | ADA, BDNF, ETNK2, EYA1, ISL1, KRT4, PALLD, PITX1, PITX2, SIM2, SOX2 |
| Tissue Development | 1.25E-05 - 3.45E-02 | 9 | BDNF, CRYAB, EYA1, PALLD, PGR, PITX1, PITX2, SIM2, SOX2 |
| Organ Development | 2.03E-05 - 3.85E-02 | 14 | ADA, BDNF, CALCRL, CRYAB, DSC2, ETNK2, EYA1, ISL1, MEIS2, PGR, PITX1, PITX2, SIM2, SOX2 |
| Organismal Development | 2.36E-05 - 2.12E-02 | 12 | ADA, ARG1, BDNF,ETNK2, EYA1, ISL1, KRT4, PGR, PITX1, PITX2, SIM2, SLPI |

*Fischer's exact test was used to calculate a p-value determining the probability that each biological function assigned to that data set is due to chance alone.

**Table S6.**

**Ingenuity Pathways Analysis: Top Bio Functions - Physiological System Development and Function for Genes Upregulated more than 10-fold in Skin Epithelium**

**Category P-value* # Molecules Molecules**

| Connective Tissue Development and Function | 1.14E-05 - 4.93E-02 | 13 | Wdnm1-like, CXCL12, EGR2, FABP4, HOXD8, HOXD10, HOXD11,LHX2, LPL, MMP3,RDH16, SCD, THBS1 |
| --- | --- | --- | --- |
| Hair and Skin Development and Function | 3.14E-05 - 4.12E-02 | 12 | ADAMTS9, DCT, ELOVL3, HOXC13, KRT25, KRT27, KRT71, KRTAP16-7, SILV, THBS1, TIMP3, TYRP1 |
| Organ Development | 6.10E-05 - 4.12E-02 | 11 | CYP1B1, GJA3, HOXC13, HOXD11, KRT25, KRT27, KRT71, KRTAP16-7, MMP3, THBS1, TYRP1 |
| Organismal Development | 1.41E-04 - 3.31E-02 | 11 | COL4A1, CXCL12, HOXA9, HOXC13, HOXD8, HOXD9, HOXD10, HOXD11, LHX2, MMP3, THBS1 |
| Nervous System Development and Function | 1.97E-04 - 4.93E-02 | 11 | ALCAM, ATP1B1, CD36, CXCL12, EGR2, GATA3, HOXD10, LHX2, POU3F1, SEMA3D,THBS1 |

*Fischer's exact test was used to calculate a p-value determining the probability that each biological function assigned to that data set is due to chance alone.
